# Perceptions and use of technology in older people with ophthalmic conditions

**DOI:** 10.12688/f1000research.17181.2

**Published:** 2019-08-05

**Authors:** Zaria C. Ali, Savana Shakir, Tariq Mehmood Aslam

**Affiliations:** 1 Central Manchester Healthcare NHS Trust, Manchester, UK; 2 University of Manchester, Manchester, UK; 3 Centre for Ophthalmology and Vision Sciences, Institute of Human Development, University of Manchester, Manchester, UK; 4 Heriot Watt University, Edinburgh, UK

**Keywords:** Ophthalmology; Perceptions; Opinions; Technology; Elderly

## Abstract

**Background:** Technologies such as mobile applications are increasingly being developed for patients to help manage their clinical conditions. However there is a paucity of information confirming the capacity or willingness of older patients with ophthalmic complaints to engage with such computer applications. The aim of this paper is to assess the perception and use of a range of common computing technologies by older ophthalmic patients, in order to guide future ophthalmology-specific development and clinical use.

**Methods:** Patients attending Manchester Royal Eye Hospital were surveyed with questions designed to measure their perceptions, attitudes and experiences of using technology.  Inclusion criteria included any patient aged 40 or over who attended the ophthalmology outpatients department.

**Results:** A total of 300 patients completed the questionnaire. The male-to-female ratio was 128:169. The majority of patients owned predominantly mobile forms of technology such as tablets and smart phones. The most common uses of technology were for communicating with friends, watching television and gathering information. Patients aged over 80 had particular difficulty using technology and used it less regularly. Less than 10% overall stated eyesight as a reason for stopping using technology.

**Conclusions:** Technology is used regularly by a large proportion of older ophthalmic patients, with numbers reducing significantly only in those aged 80 years or over. There appears to be potential for further medical use, though developers and clinicians should consider the perceptions and challenges highlighted through this survey.

## Introduction

Over the past decade there has been a surge of in the number of digital technologies aimed at assisting patients and health professionals in managing clinical conditions, including applications (apps) to help monitor chronic diseases ^1^ and social media to share experiences and information ^2^. Much of this new technology focuses on diabetes, hypertension and weight management ^1, 3– 8^, but there have been developments in a wide range of clinical conditions ^3, 9^, including ophthalmology.

One large area of interest is in apps on computer tablets or phones to monitor vision at home to benefit those with conditions such as age-related macular degeneration (AMD) ^10, 11^. Other applications include those to help educate patients on their nutrient intake or ocular condition ^12– 14^. Ophthalmic patients may have distinct characteristics relative to other clinical groups in terms of their epidemiology, clinical conditions and treatment burdens. Whilst there are many devices available and in development for ophthalmic patients, there is relatively little information on the specific needs, preferences and perceptions ophthalmic patients may have towards these emerging technologies. Although there are studies looking at how patients in general use and feel about technology ^15, 16^, there is a distinct lack of studies looking at how patients specifically with ophthalmic conditions utilise it. The importance of understanding the intended patient bases with any technology development is highlighted by those trying to integrate web based tools for those with mental health problems. Some studies have found that negative views of users lead to disuse ^17^, with target populations unwilling to engage ^18^.

The aim of this study was to assess how patients with ophthalmic conditions use technology and their attitude towards it, with a focus on older patients recruited from outpatient clinics.

## Methods

### Study background

The study took place between November 2014 and July 2015. A survey was designed that would capture basic patient demographics, type of technology used, frequency of use, how patients used technology, their views regarding how useful technology is to them and what potential barriers are to using technology. Input for the questions were derived from a patient public involvement (PPI) group where participants were asked about their views regarding technology and how they felt about using technology to help them manage their ophthalmic health. In addition input was derived from consultant ophthalmologists and experts in clinical technology use. Ethical approval for the study was given by the ethics committee of London Camden and Islington (REC reference number14/LO/1496, IRAS ID159394), and research was conducted in accordance with the Declaration of Helsinki. The ethics committee felt written consent was not needed for this study, as such verbal informed consent was taken from all participants and taken as confirmed if the participant returned their questionnaire.

The resulting survey was issued to 300 patients who attended Manchester Royal Eye Hospital outpatient clinics. Although our main focus was to determine characteristics of patients over 50 years, we decided to include some younger patients as a comparison. Inclusion criteria was any person who was a patient at Manchester Royal Eye Hospital aged over 40 years. The only exclusion criteria was the inability to understand written English. Patients were recruited on an opportunistic basis; they were approached whilst waiting for their scheduled appointment. Patients were given the questionnaire and completed it before their appointment. Patients were offered assistance in completing the questionnaire or could complete it independently. A researcher was also readily available should the patient require further clarification for any of the questions. The survey was then handed back to the researcher. In order to gain the views of those with a variety of ophthalmic conditions patients from a variety of subspecialty clinics were approached, including oculoplastics, neuro-ophthalmology, medical retina, and vitreo-retinal and glaucoma clinics.

The questionnaire and the rationale for each question is discussed below.

**Q1. Do you currently use technology to help with tasks in your everyday life, e.g. Mobile phones, computers, etc.?**

Patients could answer yes or no to this question. This was asked to give a quick and immediate insight into how many patients used technology on an everyday basis.

**Q2. Which of the following devices do you own?**

- Desktop computer
- Laptop computer
- Tablet (iPad, Nexus, Windows surface etc.)
- Smart phone (iPhone, HTC etc)
- iPod or MP3 player
- eBook Reader
- Other (Please state)

For each device patient could choose one of four options; ‘own and use’, ‘own but don’t use’, ‘plan to buy’ and ‘don’t need’. This question was designed to assess which technology patients already owned and therefore which would be the most useful to develop aids for. Discussing technology within the PPI group revealed that although patients may own computers or tablets they may not use it which may cause the results to be misleading, so the option of ‘own but don’t use’ was included. Conversely, patients may be planning to purchase these devices in the future, so to survey this potential interest the option of ‘plan to buy’ was also included. The option of ‘don’t need’ was also added to see if patients felt they were unnecessary for them.

**Q3. How often do you use technology to help with the following activities?**

- Gathering Information e.g. researching a purchase or finding a recipe
- Communicating with friends e.g. mobile phone, email, social media, online groups, Skype, texting etc
- Listening to the radio or Watching TV shows or Videos
- Finding information about your medical diagnosis, doctor, or healthcare organisation
- Booking appointments e.g. Doctors, Opticians, Dentist
- Creative pursuits – photography, music, genealogy etc.
- Playing Games
- Using online tests to test your health
- Reading
- Online learning e.g. classes to learn a language or new skill
- Planning a travel route (e.g. via Satnav or public transport)

For each activity patients could choose one of 5 options:
- Regularly (daily)
- Often (a few times per week)
- Sometimes (monthly or less)
- I know of them
- Never

Question 4 was designed to gauge how widely used technology was in our patient base, and if it was purely for leisure, practical tasks such as planning a travel route, or health related tasks such as booking appointments or looking up information. The activities outlined above are the common tasks patients mentioned they carried out using technology during the PPI meeting. If patients used technology for practical and/or health related tasks already it may be they are more open to using technology to help manage their health. Giving the option of choosing how often they used technology for the various tasks also allowed us to see how regularly they used it.

**Q4. How much difficulty do you have using technology?**

Patients could choose one of four options:

- No difficulty at all
- A little difficulty
- Moderate difficulty
- Extreme difficulty

A barrier to using technology could be difficulty in actually using it which was why this question was posed.

**Q5. How much experience do you have with the following?**

- Using Facebook
- Using phone and/or tablet apps
- Reading eBooks
- Using Twitter
- Sending email
- Browsing the internet
- Watching online TV or videos (iPlayer, YouTube etc.)
- Listening to podcasts or online radio broadcasts
- Using Skype
- Playing games
- Other (please state)

For each activity patients could choose one of four options; they could either state they were an ‘expert’, ‘amateur’, ‘novice’ or that they ‘never use’ technology for this purpose. It has previously been found that although patients may use technology on a regular basis they may perceive their ability to use technology as poor ^15^. We therefore thought it valuable to assess how they felt about using some of the most popular technology currently used, as this could affect their willingness to engage in aids utilising these platforms.

**Q6. Does anything stop you from using some of the technology we have mentioned?**

- I don't have time to learn how to use it
- My ICT skills are poor
- Devices are too complicated to learn
- My eyesight is too bad to see clearly
- Technology is not for people like me
- I am not aware of technology and what it can be used for
- It is too expensive
- It is too invasive - I don't want to use it
- It is physically too hard to use
- Other reason

Patients could choose one of the following options for each statement:

- Strongly agree
- Mostly agree
- Neither
- Mostly disagree
- Strongly disagree

This final question was included to try and ascertain what could be barriers to patient’s use of technology. This questions includes answers addressing patient’s attitudes towards technology that may be more amenable to change with good communication and user friendly and intuitive programs. It also included practical issues such as expense that may be able to be addressed with funding or by focusing on technology that patients tend to already own rather than developing completely new systems. Finally it includes physical barriers such as poor eyesight, or technology being physically too hard to use e.g. for those with severe arthritis. This may be more difficult to overcome, but could highlight the need to have other options such as enlarged text available.

## Results

### Demographics

300 patients were recruited. Male-to-female ratio was 128:169 (3 did not specify their gender). The greatest frequency of patients (44%) were in the group aged 66–79 years. Participants’ ages are summarised in Table 1.

**Table 1.** Total number of patients in each age group.

Age group	Number of patients	Percentage
40–49	8	2.7%
50–65	80	26.7%
66–79	133	44.4%
80+	79	26.3%

All were patients attending Ophthalmology outpatient clinics at the time of recruitment and 76.7% reported that they felt they had active ophthalmic problems. 32% had AMD, 14.7% had glaucoma and 4.7% were referred with cataracts. 26% did not specify what ophthalmic condition they had and answered ‘other’. 17.3% reported having no diagnosis. The raw, underlying data is available on OSF ^19^.

**Q1. Do you currently use Technology to help with tasks in your everyday life, e.g. Mobile phones, computers etc.?**

Overall 66% of patients stated they own and used technology to help with everyday tasks. Results for each age group is shown in Table 2.

**Table 2.** Percentage of patients that use technology in each age group.

Age group	% of participants in each age group that use technology,
40–49	100%
50–65	98.4%
66–79	88.5%
80+	58.6%

**Q2. Which of the following devices do you own?**

**Figure 1.**
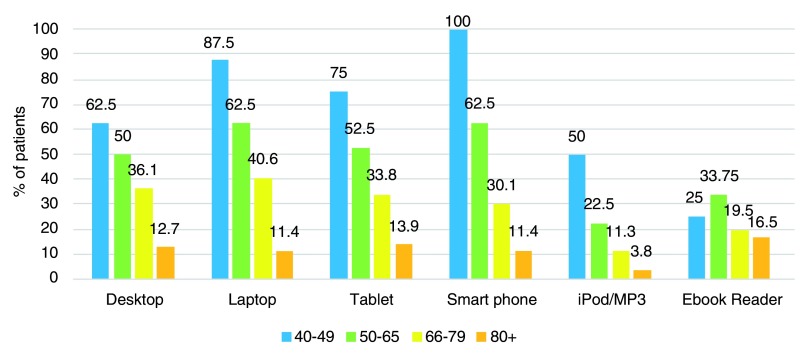
Devices owned and used by various age groups.

Results are shown in Figure 1. The four most commonly owned devices and used were smart phones, laptops and tablets. All patients aged 40 to 49 answered this question. For each device on average 18.5% of those aged 50-59 did not answer, 17.8% of those aged 60-69 did not answer and in those patients aged 80 or over 19.4% did not answer.

**Q3. How often do you use technology to help with the following activities?**

Table 3 summarises the percentage of patients who use technology for the various activities. ‘Used’ was defined as those who answered that they either did that activity regularly, often or sometimes. The age groups were able to be grouped as it was found the most commonly conducted activities were the same in all age groups, with the three most common activities being communicating with friends, watching TV, and gathering information. The results for each individual age group and for each separate response can be found in the Extended data, E1 ^20^. The main differences between age groups was a greater proportion of those under 65 stated they used technology to communicate with friends (77%) compared to those over 65 (36%). Those under the age of 65 were more likely to research their medical condition (17.5%) compared to those over the age of 65 (7.5%). Less than 10% of each age groups used technology to book appointments, do creative pursuits, do health tests or play games.

**Table 3.** Percentage of patients that used technology for various activities.

Activity	Patients who use technology for this activity, %
Gather information	60
Communicate with friends	73
Radio/TV	60.3
Find medical information	37.7
Booking appointments	24
Creative pursuits	33.7
Playing games	27.7
Health tests	5
Reading	36.7
Online learning	12
Planning a travel route	49

**Q4. How much experience do you have with various types of technology?**

Overall, participants were most comfortable sending emails and browsing the internet. The results for all age groups highlighting the two responses ‘expert’ and ‘never use’ are summarised in Table 4.

**Table 4.** Percentage of patients who stated they were either an expert at/never did various activities.

Activity	Response, %	
	Expert	Never use
Facebook	8.3	70.7
Apps	19.0	41.3
Ebooks	18.7	66.0
Twitter	1.3	89.7
Email	36.7	36.3
Browsing internet	34.7	34.0
Online TV/radio	13.3	63.3
Podcasts	6.3	76.3
Skype	9.7	65.0
Games	10.0	51.0

The results for all responses for each age group for each response can be found in the Extended data, E2 ^21^. In age groups below the age of 80, at least a third of all patients stated they were experts at these two activities. In those aged over 80, less than 10% cited feeling they were an expert in the use of any type of technology.

The majority stated they did not use social media such as Facebook (70% overall did not use it) and twitter (90% overall stated they did not use it).

**Q5. How much difficulty do you have using technology?**

**Figure 2.**
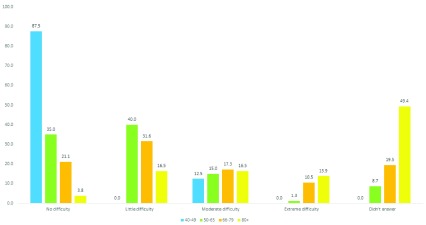
Degree of difficulty using technology in each patient group.

The degree of difficulty experienced by patients is shown in Figure 2.

From the age of 50 onwards, a majority of patients felt they had difficulties with using technology. This clearly increased with increasing age, with 13.9% of those over the age of 80 having extreme difficulties. 

**Q6. Does anything stop you from using some of the technology we have mentioned?**

**Figure 3.**
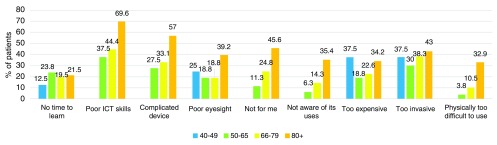
Percentage of patients who agree with various statements regarding barriers using technology.

Results of more specific barriers to using technology are summarised in Figure 3.

## Discussion

This survey provides detailed information on both the levels of technology use by older ophthalmic patients and the challenges they face, highlighting differences between different age groups.

Use of technology is ubiquitous amongst age groups of 40–49 years in their everyday lives. It is interesting to note this remains at a near-universal level till the ages of 66 years and above, and even then it drops a small amount to 88.5%. A more significant drop to 58% is seen only once the age group of over 80s is reached. The association between likelihood of using technology and age has been demonstrated previously in a survey of 1371 cancer patients where a significant correlation between increase in age and likelihood of using technology was found ^16^.

Although the uptake and use of technologies for older age groups, especially in participants over the age of 80 tends to reduce, there is no indication that this is perceived as due to inherent incapability or levels of expressed difficulty. Causes that were advocated as challenges were those that could potentially be addressed by education, with poor IT skills and over-complex devices cited as key barriers. Poor vision, expense and invasiveness were less important.

Most of the activities that patients engage in using technology are not connected to their medical condition, including watching TV and communication with friends, especially with the relatively younger ages (under 65). Although a large proportion of patients utilised technology to gather information, less than 10% overall cited using technology to research their condition. It has been found that issues with using technology to research medical conditions could be connected to how trustworthy patients perceive the source to be ^8^. Technology which has been advocated and developed by health professionals and has verified information could be highly valued ^22^. Universally patients rarely used technology to book appointments or to do health tests which may be due to a lack of health services offering this service. The ability to do this could be valued, if not by the patient but by their carers, as demonstrated by a qualitative study whereby carers felt they benefited from the use and access of a health care portal ^23^. There is clearly further scope in older patients for using the widespread ownership and ability to use portable devices for medical purposes such as patient education, interaction or monitoring. This may need at least initially to involve use of emails and websites. Developments which may not be as useful may be those revolving around social media. Although many reported regularly using technology to communicate with friends, patients generally did not feel as confident using common platforms such as Facebook and Twitter. Although social media has previously been found to help share information and experiences in those with HIV ^2^, this sort of tool may not be the most appropriate for this patient group as they may not engage. The disinterest in communicating with others was also found by Girault et al., where only 54% cited communicating with peers as important ^16^. This was an interesting contrast to other studies, where a feeling of connecting with others was one of the more positive elements of the technology related intervention ^2, 4^.

The most commonly owned technologies are portable devices; smart phones, laptops and tablet computers. Development of technologies on these devices would allow them to be accessible to a majority of patients up to the age of 66. They therefore could easily access programmes such as apps. which in turn could help improve adherence to treatment regimens ^4^ or help monitor micro-nutrient intake to help prevent the development and progression of conditions such as AMD ^24, 25^. However, beyond this age, computer device ownership drops appears to drop. Programmes to help ophthalmic patients with apps on such devices might need to incorporate distribution of such devices to older generations. In ophthalmic patients, written text is more likely to be accessible if designed for tablet computers and smart phones than e-book readers, which, despite their potential for use in the ophthalmic community, are still less popular than smart phones or tablets.

In general the uptake and use of technological devices by older patients is high. It drops in patients over the age of 80, but it is likely that this can at least be combatted by better education and training and targeting software to devices in widespread use. 

Comfort and confidence using technology appears to be an important obstacle to take into account when developing new technology for older ophthalmic patients. The younger patient groups who cited using technology regularly were also the ones who stated minimal difficulty using it. Comparatively those aged 66 and over were more likely to report having difficulty using technology and felt their ICT skills were poor despite using it regularly. This was also found in a previously published survey of 255 older patients in the community aged 60 or over ^15^. If patients perceive the technology to be difficult to use they may not use it particularly as over a third of all patient groups felt technology was too invasive and nearly a half of those over 80 feeling technology ‘wasn’t for them’. 

Ensuring any new technology developments are explained properly and ensuring patients have a source of support to help them understand and troubleshoot any potential problems could help overcome any trepidation in using technology. In all age groups bar those over the age of 80, at least a third cited being ‘experts’ at sending emails and browsing the internet, so technology support could be based online either via e-mails or a help forum. Furthermore the majority did not agree with the sentiment that finding time to use technology was a drawback which suggests that they may be willing to invest time to learn to use it. Indeed patients are more likely to use technology once they are familiar and comfortable with it ^26^.

Another issue is expense, as a notable proportion of all age groups felt this was a barrier to technology use. Creating technology which focuses on what patients currently own could increase the likelihood of patients engaging with it. It is also important to be aware that technology cannot completely replace a health professional and patients have frequently cited the importance of in-person communication ^1, 4, 23, 27^.

Our study has a relatively large sample size, with the majority suffering from ophthalmic conditions so we can be confident that the findings are reflective of this patient group. However there are limitations to our study. Although most had ophthalmic conditions, nearly a quarter of patients felt they did not. This is likely due to the fact that these patients attended for follow up at a general clinic following an acute eye problem.

We also did not explore other demographic data which may explain how patients use technology. Our study did not gather data on ethnic origin or social class, which might have provided further useful information for application development. For example, a study exploring the use of a health portal system found that those with lower health literacy and ethnic minorities are less likely to use health portals ^23^. If this is similar in our patient base it may suggest that additional support such as the availability of alternative languages may be required. It may also have been of interest to enquire as to whether they lived alone as one study looking at the acceptability of e-health interventions in chronic pain found those who were older and lived alone were more likely to use technology ^28^. It may be that those who were older in our patient group already had family or relatives to support them nearby, so did not feel the need to use technology to help them.

In our survey participants often didn’t answer questions which may have skewed results; for example when answering question 4 they may not have answered sections as they did not use technology for that particular activity, meaning the answer should have actually been ‘never’. There may also have been some difficulties understanding the difference between the options of ‘amateur’ and ‘novice’ in question 6. If further studies were carried out it may be of benefit to have the researcher sit and complete the questionnaire with the participant to answer any queries. Indeed this was done with 55 patients and resulted in these questionnaires being completed fully. It appears younger patients answered questionnaires more fully, so more additional support may only be required for older patients.

 Exploring the attitudes of health care providers towards using technology with patients could also be important. A comparative survey of 1406 health providers and 1102 ‘consumers’ found that the consumers were more supportive of new medical technology ^9^. It would be worth finding out the perspective of ophthalmic health professionals in using technology with their patients in daily practice as they would be the ones facilitating their use. Any new technology which is developed should also involve relevant health professionals as a mismatch between perceived benefit and applicability may affect its use in the clinical setting ^29^.

## Conclusion

Overall our patients were found to have and to use predominantly portable devices such as smart phones and tablets suggesting that new technology using these mediums could be easily accessed. Although the majority regularly use technology, many still feel under-confident with new technologies and may not perceive it as beneficial to them particularly in those aged over 65. It is therefore important that the benefit of any new technology is explained clearly to the intended patient base, is individualised, and patients carefully instructed in its use with access to support should they need it. Further studies looking at other potential barriers to using technology in detailed socioeconomic and cultural groups may be of use and it may also be of value to collect health professionals’ views towards using technology with their patients.

## Data availability

### Underlying data

Original data is available via figshare under the title ‘Original data MANAGER1’. DOI: https://doi.org/10.6084/m9.figshare.7358987.v1 ^19^.

### Extended data

Supplementary material 1. Graphs depicting full results for question 3: ‘How often do you use technology to help with the following activities?’ Extended data for MANAGER1. DOI: https://doi.org/10.6084/m9.figshare.7358969.v1 ^20^.

Supplementary material 2. Graphs depicting full data for question 4: How much experience do you have with various types of technology? Extended data for MANAGER1, part 2. DOI: https://doi.org/10.6084/m9.figshare.7358984.v1 ^21^.
