# Supplementary material for: Perceptions and use of technology in older people with ophthalmic conditions
Source: F1000Res. 2019 Aug 5;8:86. Originally published 2019 Jan 22. [Version 2] doi: 10.12688/f1000research.17181.2 (PMC6685452; doi:10.12688/f1000research.17181.2)
Supplement: Peer review history

# Peer review: Reviewer response for version 2

**DOI:** 10.5256/f1000research.21870.r52039

**Published:** 2019-08-06

**Authors:** Jill Waalen

**Affiliations:** 1 Scripps Research Translational Institute, La Jolla, CA, USA; 2 General Preventive Medicine Residency Program, University of California San Diego, La Jolla, CA, USA

The authors have addressed most of my concerns. The remaining issue is the reporting of non-response for each question. The tables and graphs are simple enough that reporting of the actual numbers (numerator and denominator  instead of just %) should be do-able and would be informative.

I confirm that I have read this submission and believe that I have an appropriate level of expertise to confirm that it is of an acceptable scientific standard.

# Peer review: Reviewer response for version 1

**DOI:** 10.5256/f1000research.18784.r43423

**Published:** 2019-05-21

**Authors:** Michel Michaelides

**Affiliations:** 1 UCL Institute of Ophthalmology, University College London (UCL), London, UK

This is a very valuable addition to the literature which is timely and needed. It is well planned and executed and should be highly cited and encourage further development in this avenue of research. There is increasing recognition that technology has a central role to play in improving visual function and such studies are an integral part of this process.

- Is the study design appropriate and is the work technically sound?

The study involves a straightforward survey of patient responses to questions on use of technology. It is not a validated questionnaire , but does not claim to be so. It uses entirely appropriate survey methodology to acquire information on patient perceptions of technology use. 

-     Are sufficient details of methods and analysis provided to allow replication by others?

The methods of survey collection are described and the questions also provided. The responses to these questions are provided in enough detail to provide the reader with a snapshot of patient perceptions. It should be noted that this cannot necessarily be translated to all populations globally and the paper accepts this. The validity is limited to the population under question and  is not necessarily transferable to other populations. It is however still very useful information as real world data a patient population in a typical teaching hospital in U.K 

 There is very little existing information on this topic and therefore this paper is very important in informing doctors and developers on patient attitudes to technology.

I confirm that I have read this submission and believe that I have an appropriate level of expertise to confirm that it is of an acceptable scientific standard.

# Peer review: Reviewer response for version 1

**DOI:** 10.5256/f1000research.18784.r45508

**Published:** 2019-03-25

**Authors:** Jill Waalen

**Affiliations:** 1 Scripps Research Translational Institute, La Jolla, CA, USA; 2 General Preventive Medicine Residency Program, University of California San Diego, La Jolla, CA, USA

The study describes the results of a survey of 300 patients age 40 years and older attending the Manchester Royal Eye Hospital regarding their use of technology and reasons for not using it. While the use of a simple and short apparently non-validated questionnaire along with a convenience sample limits the value of the results, it nonetheless provides data in an area that is currently underrepresented in the literature, i.e., how technology may be accepted and used in populations with special considerations, like limited eyesight.

 Most of the following specific suggestions for improvement are minor: 

1. Methods, paragraph 2:  More details are needed to described the administration of the questionnaire (some of which are actually alluded to in the Discussion), including whether the questionnaire had to be completed on site (and before seeing a provider) and whether help was systematically available to the survey takers. Also, with such a variety of ages and conditions, it would have been helpful to have some measure of eyesight impairment. Was there any attempt to ascertain this? 
2. Results, paragraph 1: Gender numbers do not add up. 169 male + 127 female + 3 did not specify =299 (not 300).
3. Results, paragraph 1: It is stated that 44% of patients were in the 66-79 years age category. It would be helpful to give the full breakdown by age, with n’s included, perhaps in Table 1 (which is entitled “Age of patients”, but in fact is "Use of Technology by Age Category”). 
4. Results, graphs: Although the graphs are an efficient way to convey the results (and tends to be preferred by readers) compared with tables, given the comment in the Discussion that participants often didn’t answer questions, which may have skewed results), it would be helpful to have the denominators for the percentages reported in the graphs.  

 Minor editorial comments:

1. Discussion, paragraph 3: “the over 80s” should be changed to “participants over age 80” or something less colloquial.
2. Discussion, paragraph 9: would add “relatively” to the description of the sample size as large.

I confirm that I have read this submission and believe that I have an appropriate level of expertise to confirm that it is of an acceptable scientific standard, however I have significant reservations, as outlined above.

# Response

**Published:** 2019-07-08

**Authors:** Zaria Ali

**Affiliations:** Central Manchester Healthcare NHS Trust, UK

**Response to reviewer**

Many thanks for your review. Please find a summary of the changes made in response to your comments.

1. Methods, paragraph 2: More details are needed to describe the administration of the questionnaire (some of which are actually alluded to in the Discussion), including whether the questionnaire had to be completed on site (and before seeing a provider) and whether help was systematically available to the survey takers. Also, with such a variety of ages and conditions, it would have been helpful to have some measure of eyesight impairment. Was there any attempt to ascertain this?

**The methods have been amended to state when the patients had to complete the questionnaire (before their appointment) and the availability of help (patients aware researcher nearby should they require assistance, but also had the option to sit and complete the questionnaire with a researcher should they prefer). Unfortunately we did not have an item on the questionnaire which would ascertain the level of visual impairment, although we agree this could have been helpful, and would be helpful to include in future studies.**

2. Results, paragraph 1: Gender numbers do not add up. 169 + 127 + 3 did not specify =299 (not 300).

**The data has been reviewed and the numbers have been amended (should be 128 not 127).**

3. Results, paragraph 1: It is stated that 44% of patients were in the 66-79 years age category. It would be helpful to give the full breakdown by age, with n’s included, perhaps in Table 1 (which is entitled “Age of patients”, but in fact is "Use of Technology by Age Category”).

**The tables have been rearranged to be clearer, and as suggested we have included the actual number of patients in table 1 as opposed to just percentages.**

Results, graphs: Although the graphs are an efficient way to convey the results (and tends to be preferred by readers) compared with tables, given the comment in the Discussion that participants often didn’t answer questions, which may have skewed results), it would be helpful to have the denominators for the percentages reported in the graphs.

**Thank you for your comment. We agree that it is useful to assess the percentage of patients for each age group that did not answer. For figure 1 we felt that adding the percentage of each age group for each device would complicate the graph and make it more confusing, as such it is written in text instead.** **For figure 2 it was possible to easily demonstrate what percentage of patients in each age group did not answer. For Figure 3 all patients answered this questions so the graph has been left unaltered**

**Minor editorial comments:**

1. Discussion, paragraph 3: “the over 80s” should be changed to “participants over age 80” or something less colloquial.

**This has now been changed, and also been changed further down in the discussion as well.**

1. Discussion, paragraph 9: would add “relatively” to the description of the sample size as large.

**This has now been added.**
